# A Cautionary Note on the Effects of Population Stratification Under an Extreme Phenotype Sampling Design

**DOI:** 10.3389/fgene.2019.00398

**Published:** 2019-05-03

**Authors:** Michela Panarella, Kelly M. Burkett

**Affiliations:** ^1^Department of Biology, University of Ottawa, Ottawa, ON, Canada; ^2^Department of Mathematics and Statistics, University of Ottawa, Ottawa, ON, Canada

**Keywords:** association study, extreme phenotype sampling, population stratification, principal component analysis, Type 1 error

## Abstract

Extreme phenotype sampling (EPS) is a popular study design used to reduce genotyping or sequencing costs. Assuming continuous phenotype data are available on a large cohort, EPS involves genotyping or sequencing only those individuals with extreme phenotypic values. Although this design has been shown to have high power to detect genetic effects even at smaller sample sizes, little attention has been paid to the effects of confounding variables, and in particular population stratification. Using extensive simulations, we demonstrate that the false positive rate under the EPS design is greatly inflated relative to a random sample of equal size or a “case-control”-like design where the cases are from one phenotypic extreme and the controls randomly sampled. The inflated false positive rate is observed even with allele frequency and phenotype mean differences taken from European population data. We show that the effects of confounding are not reduced by increasing the sample size. We also show that including the top principal components in a logistic regression model is sufficient for controlling the type 1 error rate using data simulated with a population genetics model and using 1,000 Genomes genotype data. Our results suggest that when an EPS study is conducted, it is crucial to adjust for all confounding variables. For genetic association studies this requires genotyping a sufficient number of markers to allow for ancestry estimation. Unfortunately, this could increase the costs of a study if sequencing or genotyping was only planned for candidate genes or pathways; the available genetic data would not be suitable for ancestry correction as many of the variants could have a true association with the trait.

## 1. Introduction

Extreme phenotype sampling (EPS)—also called selective genotyping, trait or outcome dependent sampling—is a popular study design for increasing the power of genetic association studies. Assuming a large cohort with continuous phenotype data is available, EPS involves only genotyping individuals in the top and bottom extremes of the phenotype distribution. The rationale for this design is that the phenotypic extremes are enriched for either deleterious or protective variants (Kryukov et al., 2009) and so the power to detect genetic effects can be maintained even while genotyping a smaller subset of a larger cohort (Lander and Botstein, 1989; Van Gestel et al., 2000; Kryukov et al., 2009; Guey et al., 2011; Barnett et al., 2013).

The EPS design has been used in the genetic mapping context for some time. Lander and Botstein (1989) described how this design could be used in linkage analysis to map QTLs. Darvasi and Soller (1992) examined optimal proportions to genotype and they demonstrated that for linkage studies it is not useful to genotype more than the lower and upper 25*th* percentile. The EPS design has been used in candidate gene association studies (Morabia et al., 2003; He et al., 2006; Sims et al., 2008; Boora et al., 2016) and in genome-wide association studies (Versmissen et al., 2015). More recently, EPS has been proposed as an approach to increase power of rare variant and exome sequencing studies at a fixed sample size. For example, Barnett et al. (2013) showed that EPS has higher power to detect rare variants when compared to random sampling. The EPS design has been applied to whole-exome sequencing studies in order to find cystic fibrosis modifier genes (Emond et al., 2012), variants associated with pulmonary disease (Bruse et al., 2016), and with diabetic retinopathy (Shtir et al., 2016).

Although specialized methods accounting for the extreme sampling have been developed (Slatkin, 1999; Wallace et al., 2006; Huang and Lin, 2007; Liu and Leal, 2012; Barnett et al., 2013; Lin et al., 2013), a simple analysis strategy of treating extreme group membership as a binary trait is common (for example, Emond et al., 2012; Zhou et al., 2016). Conventional methods such as a Pearson chi-square test or logistic regression can be applied to determine if group membership is associated with genotype; specialized methods and software are not required to compute these statistics. This analysis strategy is valid, though not optimal (Lin et al., 2013), as the underlying continuous trait values are effectively ignored after the subsampling.

As outlined above, there has been substantial work in investigating the power of these designs for gene mapping and in developing statistical methods that account for the extreme sampling. However, there is little information available about the confounding effects of population stratification when this study design is used. Population stratification is known to cause an increase in the rate of false positive associations for both case-control studies and for quantitative trait analysis (Haldar and Ghosh, 2012). However, the extent to which confounding is a concern for EPS designs has not been established. Guey et al. (2011) suggested that EPS could inflate confounding due to population stratification, but they did not investigate the extent of the inflation. Lin et al. (2013) investigated the bias due to population stratification when the phenotype values were erroneously analyzed using linear regression. They did not evaluate the confounding effect when the data is analyzed as a dichotomous trait.

Multiple approaches have been developed to correct for ancestry in association studies, including Genomic Control (Devlin and Roeder, 1999), STRUCTURE/STRAT (Pritchard et al., 2000), Principal Components/EIGENSTRAT (Price et al., 2006), and Linear Mixed Models (Kang et al., 2008, 2010; Lippert et al., 2011; Listgarten et al., 2012; Zhou and Stephens, 2012). In particular, principal component (PC) based corrections are widely used when genome-wide data is available. For example, in their study utilizing EPS, Emond et al. (2012) adjusted for ancestry by including the top 3 PCs in a logistic regression model. However, there have also been published associations using EPS designs where ancestry correction was limited to self-identified ethnicity data. For example, in a replication study described in Herbert et al. (2006), Polish and American samples were combined and a previously associated SNP was genotyped in both the upper and lower extremes of BMI. Analysis was stratified in the two groups, but no correction for hidden ancestry was done. Boora et al. (2016) describe a recent candidate gene study where an EPS design was used; the statistical analysis did not include correction for ancestry though they did ensure that the two groups were balanced in terms of proportion of Caucasians and African Americans.

In addition to the quantification of the effects of confounding under EPS, it is important to verify that existing methods developed for quantitative traits or case-control studies also work in the EPS setting. McVean (2009) demonstrated that uneven sampling of underlying population can distort PC estimation. When there is confounding due to population stratification, EPS could result in unequal sampling from the underlying populations. The effect that this could have on a PC-based correction is unknown. We assume that PCs would provide the same correction in EPS as in case-control sampling; however, we are not aware of work confirming this assumption. In addition, PC and linear mixed model approaches to correct for confounding from population stratification with common variants may not provide the same correction for rare variants (Mathieson and McVean, 2012; Persyn et al., 2018). As in the common variant case, neither the effects of confounding when the candidate SNPs are rare nor a PC-based correction have been evaluated under EPS sampling schemes.

The purpose of this work is 2-fold. We first evaluate whether EPS inflates the false positive rate due to population stratification. Using extensive simulations, we demonstrate that the false positive rate under the EPS design is significantly higher than a random sample of the same size from the same cohort when the data is analyzed using the simplest method of analysis (a Pearson chi-square test or ANOVA). The false positive rate is also higher than a “case-control” like design where the cases are from one extreme of the phenotype and the controls randomly sampled. We also show that the false positive rate increases with sample size and that we can expect an inflated false positive rate even with parameter values selected to model European population data. Using the procedure in Emond et al. (2012) as a model analysis, we then verify that the widely-used PC based correction adequately controls the type 1 error rate for common variants. We conclude that population stratification correction is especially important under the EPS design and that any studies utilizing this sampling design must ensure that genotyping includes a sufficient number of markers for ancestry estimation.

## 2. Methods

Assume that we have large cohort comprised of two hidden subpopulations, and that the phenotype of interest is normally distributed within each subpopulation:

(1) $$\begin{array}{r} Y_{ij}\sim N\left(\mu_{i},\sigma^{2}\right),j=1\ldots n_{i} \end{array}$$

where *Y*_*ij*_ is the observed phenotype of the *jth* individual in population *i*, *i* = 1, 2, and the sample size from the *ith* population is *n*_*i*_. For simplicity, we assume that the phenotypic mean and variances are μ_1_ = −μ_2_ and σ^2^ = 1, respectively. Let ω_*i*_ be the proportion of the cohort from subpopulation *i* and so ω_1_ + ω_2_ = 1. The distribution of *Y* in the cohort, not conditional on population, is a mixture of two normal distributions:

(2) $$\begin{array}{r} F(y)=\omega_{1}\Phi \left(\frac{y-\mu_{1}}{\sigma }\right)+\omega_{2}\Phi \left(\frac{y+\mu_{1}}{\sigma }\right) \end{array}$$

where Φ is the CDF of the standard normal distribution.

We will assume that we are testing for association between the phenotype and a candidate SNP with alleles labeled *A* and *a*. Let *p*_*i*_ be the probability of allele *A* in the *ith* subpopulation and assume that within subpopulation the genotype frequencies follow Hardy-Weinberg Equilibrium (HWE). Therefore, the genotype probabilities in the combined population are

(3) $$\begin{array}{lll} p_{AA} & = & \omega_{1}p_{1}^{2}+\omega_{2}p_{2}^{2};\;p_{Aa}=2\omega_{1}p_{1}\left(1-p_{1}\right)+2\omega_{2}p_{2}\left(1-p_{2}\right);\\ p_{aa} & = & \omega_{1}{\left(1-p_{1}\right)}^{2}+\omega_{2}{\left(1-p_{2}\right)}^{2}. \end{array}$$

The expected genotype counts are obtained by multiplying the genotype probabilities by the size of the cohort, *N*. We will assume that under EPS those in the top and bottom 10% of the phenotype distribution are genotyped; the sample size for each group is *n* = 0.1*N*.

### 2.1. Estimating the False Positive Rate Under EPS and Random Sampling

To assess the false positive rate due to confounding from population stratification, we assume that conditional on population membership there is no true association between the candidate SNP and the phenotype. The phenotype distribution within each genotypic category can also be written as a mixture distribution of the two normal components:

$$\begin{array}{l} F\left(y|g=AA\right)=\mathrm{Pr}\left(i=1|g=AA\right)F\left(y|i=1,g=AA\right)\\ \;+\mathrm{Pr}\left(i=2|g=AA\right)F\left(y|i=2,g=AA\right)\\ \;=\frac{\omega_{1}p_{1}^{2}}{\omega_{1}p_{1}^{2}+\omega_{2}p_{2}^{2}}\Phi \left(\frac{y-\mu_{1}}{\sigma }\right)\\ \;+\frac{\omega_{2}p_{2}^{2}}{\omega_{1}p_{1}^{2}+\omega_{2}p_{2}^{2}}\Phi \left(\frac{y+\mu_{1}}{\sigma }\right) \end{array}$$

(4) $$\begin{array}{rc} \;= & p_{1|AA}\Phi \left(\frac{y-\mu_{1}}{\sigma }\right)+p_{2|AA}\Phi \left(\frac{y+\mu_{1}}{\sigma }\right) \end{array}$$

(5) $$\begin{array}{rcl} F\left(y|g=Aa\right) & = & p_{1|Aa}\Phi \left(\frac{y-\mu_{1}}{\sigma }\right)+p_{2|Aa}\Phi \left(\frac{y+\mu_{1}}{\sigma }\right) \end{array}$$

(6) $$\begin{array}{rcl} F\left(y|g=aa\right) & = & p_{1|aa}\Phi \left(\frac{y-\mu_{1}}{\sigma }\right)+p_{2|aa}\Phi \left(\frac{y+\mu_{1}}{\sigma }\right) \end{array}$$

where *p*_*i*|*g*_ denotes the probability of being in population *i* given genotype *g*. These conditional probabilities are easily found by substituting the relevant expression from Equation (3) in to the expression for the conditional probability: Pr(*i*|*g*) = Pr(*g*|*i*)Pr(*i*)/Pr(*g*).

Population stratification is known to be a confounding factor in population-based genetic association studies where the cohort is a random sample of the population and the phenotype is quantitative (Haldar and Ghosh, 2012). Therefore, it is important to compare the false positive rate under EPS to the rate for equally-sized random samples from the full cohort. For a quantitive phenotype, the null hypothesis of equal phenotypic mean across the different genotypic classes can be tested with *t*-tests (dominant, recessive models), ANOVA (codominant model) or linear regression (additive model). All assume that the phenotype is normally distributed with equal variance, conditional on the genotypic class. Equations (4)-(6) show that this assumption is violated when there is population stratification. Nevertheless, we can compute the *F* or *t*-test statistics as tests of association, but their sampling distributions will not be the usual *F* or *t* distributions since under both the null and alternative hypotheses the phenotype conditional on genotype is not a simple normal distribution. Because phenotype conditional on genotype is not normally distributed, we cannot use the non-central *F* distribution to compute the probability of rejecting the null hypothesis when the means are not equal. This makes it difficult to analytically compute the false positive rate under random sampling and we therefore proceed by simulation. Note that under the EPS design, we can compute the false positive rate analytically. However, since our goal is to compare the rates between the EPS and random design, we have used the same simulations to estimate both rates. The method for analytically computing the false positive rate for the EPS design is given in the Supplementary Material.

We use simulation to estimate the false positive rate for the random sampling, case-control like sampling and EPS designs. We assume a phenotyped sample of *N* = 5, 000 individuals that is a mixture of the two subpopulations, as described above. For particular parameter values (*p*_1_, *p*_2_, μ_1_, ω_1_) we simulate genotype data within each subpopulation with genotype frequencies under HWE and phenotype data from the normal distributions given in Equation (1). Note that the genotypes and phenotypes are simulated independently; therefore, the genotype is not causally associated with the phenotype. The parameter values chosen for these simulations are provided in Table 1.

**Table 1:** Genetic and phenotypic parameter settings for simulations to assess confounding effect of population stratification.

**Parameter**	**Description**	**Values**
N	Full cohort sample size	5000
n	Sample size for each extreme	0.1N = 500
*w*_1_	Proportion of full sample from population 1	0.3, 0.4, 0.5, 0.6, 0.7
*w*_2_	Proportion of full sample from population 2	1 − *w*_1_
*p*_1_	Frequency of A allele in population 1	0.5 to 0.9, by 0.1
*p*_2_	Frequency of A allele in population 2	0.5 to 0.9, by 0.1
μ_1_	Phenotype mean in population 1	0.1, 0.2

After the data for the full cohort has been simulated, the subsamples are drawn. For the EPS design, the highest 500 individuals and lowest 500 individuals in the phenotype distribution are selected, which corresponds to the 10% extremes. For random sampling, we simply randomly sample 1,000 individuals from the full cohort. To simulate the case-control type design, we labeled the 500 individuals from the top extreme as cases, and we randomly sampled the controls from the remaining 4,500 individuals in the cohort. This would correspond to a trait where an individual is considered to have a disease if a quantitative variable exceeds a threshold.

We evaluated a codominant, an additive and a recessive disease/trait model. Genotype is categorized as either (AA, Aa, aa), (0,1,2), or (AA+Aa,aa) for the codominant, additive and recessive models, respectively. For the EPS and case-control samples, the data is cross-classifed on genotype and upper/lower (EPS) or upper/random (case-control) group status. A Pearson chi-square test, a difference of proportions test, or Cochran-Armitage test is applied for the codominant, recessive and additive disease models, respectively. For the random sample, genotype is categorized as for the EPS samples and either ANOVA (codominant, recessive) or linear regression (additive) is performed.

For each combination of parameter values, we simulated 10,000 datasets and determined whether the null hypothesis of no association would be rejected at level α = 0.05. The proportion of the 10,000 datasets where the null is rejected is an estimate of the false positive rate.

We also included limited simulation scenarios where the candidate SNP was not a common variant. In particular, the simulations described above were run with a minor allele frequency (MAF) for the candidate SNP of 0.01 in population 1 (rare variant) and either 0.01, 0.05 (low frequency variant) or 0.10 (common variant) in population 2.

Finally, although we selected a wide range of parameter values, we also wanted to ensure that the parameter values were realistic. We therefore also computed false positive rates using parameter values inspired by European population data. We simulated two populations with allele frequencies set to the frequency of the lactose tolerance variant in Italy and France (0.286 for Italy and 0.43 for France) (Sahi, 1994) and using data for female height as our phenotype variable (means of 158.48 cm for Italy and 161.77 for France, both variances set to 6) (Onland-Moret et al., 2005). We chose the lactose gene since it is known to vary between European populations (Campbell et al., 2005).

All analysis was completed in R (http://cran.r-project.org/); the Cochran-Armitage test was performed using the R package DescTools (Signorell et al., 2018).

### 2.2. Assessment of PC-Based Correction for Confounding

We also performed limited simulations to verify that adjustment of the logistic regression model with the top principal components (PCs) controls the type 1 error under an EPS study design. For all the scenarios for evaluating the PC-based correction, our parameter values were chosen to simulate a worst-case situation where confounding due to population stratification would be high. If the PC-based correction performs well under the worst case scenarios, then we would expect it would work when the sample consists primarily of only one subpopulation or the allele frequency differences between the subpopulations were quite small.

We simulated a cohort of *N* = 5, 000 individuals consisting of two subpopulations of equal size (ω_1_ = ω_2_ = 0.5). The phenotype values were again sampled from a normal distribution within each subpopulation with means μ_1_ and μ_2_ = −μ_1_. We varied the values for μ_1_ (given in **Table 4**). To simulate our candidate SNP, we set the “A” allele frequencies to be *p*_1_ = 0.5 and *p*_2_ = 0.9 in the two subpopulations and sampled genotypes assuming HWE within each subpopulation. This represents an extreme difference in allele frequency for the candidate SNP.

For ancestry correction, we use genotype data on a large number of SNPs. In practice, we would use genome-wide SNPs and the majority of these SNPs would not be expected to be associated with the trait. To simulate our ancestry SNPs, we first used the Balding-Nichols model (Balding and Nichols, 1995), which was also the approach used in Price et al. (2006). For each dataset, we simulated genotype data on 5,000 SNPs as follows. For a given marker, a generating allele frequency, *p*, for the “1” allele was sampled from a uniform (0.1,0.9) distribution. For each of the two subpopulations, their allele frequency was sampled from a beta distribution with parameters α = *p*(1 − *F*_*st*_)/*F*_*st*_ and β = (1 − *p*)(1 − *F*_*st*_)/*F*_*st*_, where *F*_*st*_ is the fixation index capturing population differentiation. We set *F*_*st*_ = 0.01, which is higher than would be expected between continental European populations (Nelis et al., 2009). The genotype for the *ith* SNP and *jth* individual, *g*_*ij*_, was sampled from a multinomial distribution with probabilities determined by assuming HWE and using the subpopulations allele frequency.

Following Price et al. (2006), we centered and scaled each ancestry SNP genotype by subtracting the mean genotype across both groups and dividing by $\sqrt{\tilde{p_{i}}\left(1-\tilde{p_{i}}\right)}$ where

$$\tilde{p_{i}}=\frac{1+\sum_{k=1}^{n}g_{ik}}{2+2n}$$

is an estimate of the frequency of the “1” allele for the *ith* SNP. The summation is taken over all *n* individuals in the subsample. We then computed the principal component scores for the top five principal components using the prcomp() function in R.

Logistic regression was used to test for association between the candidate SNP and our extreme phenotype categories. The disease model was assumed to be either codominant (genotype coded as a factor) or additive (genotype coded as a numeric variable). The model adjusting for ancestry included fixed effects for each of the top five PCs. The proportion of simulated datasets where a likelihood ratio test of association between group status and candidate SNP genotype gives a *p*-value less than α = 0.05 was used to estimate the false positive rate. As the data was generated to have no true causal association between candidate SNP and phenotype, we would expect our estimate to be close to 0.05 if the type 1 error rate is controlled. For each different value of μ_1_, a total of 2,000 simulations were performed.

Although the Balding-Nichols approach will generate allele frequency differences that give the desired *F*_*st*_ value, the data may not produce genotype data similar to human genotype data. For this reason, we also ran a scenario where publicly-available genotype data from the 1,000 Genomes project (Phase 1) (The 1000 Genomes Project Consortium, 2015) was used as reference data for simulating ancestry SNPs. We used data from the unrelated individuals in the European-derived populations consisting of the British (GBR), Finnish (FIN), Spanish (IBS), and Italian (TSI) samples. We randomly sampled 200,000 SNPs having MAF >0.05 from across the 22 chromosomes. For each SNP, we computed the major allele frequency in each subpopulation. We removed any SNPs where the minor allele was not observed in one of the four subpopulations and where the maximum allele frequency among all four populations was less than 0.1, which left 158,781 SNPs.

We then generated a cohort of size *N* = 5, 000 with equal proportion coming from each of the four subpopulations. Phenotype data was simulated within each subpopulation from a Normal distribution with unit variance and mean vector μ = (0.3, 0.2, 0, −0.1) for the Italian (TSI), Spanish (IBS), UK (GBR) and Finnish (FIN) samples, respectively. We excluded all but the top and bottom 10% of the sample and stored population of origin. The ancestry SNP data was generated for each of the approximately 150,000 SNPs using the estimated allele frequency for the subpopulation and assuming HWE. Finally, we selected the candidate SNP from those SNPs where the −log_10_ of the *p*-value from a Fisher exact test of equal allele frequencies across subpopulations was greater than 4. This ensured that there would be confounding due to population stratification. For each dataset, we estimated principal components using the centered and scaled genotype data. Due to the large size of the genotype dataset, the function bigcor() from the propagate package (https://cran.r-project.org/web/packages/propagate/index.html) was used to compute the covariance matrix; PCs were then calculated using output from the eigen() function. Logistic regression was used to test for association between group status (high or low phenotype) and candidate SNP genotype in models both with and without the top five PCs. Simulating the ancestry SNPs and performing the principal component decomposition was computationally intensive and so 1,000 simulations were performed for this scenario.

Finally, we also investigated the PC-based correction when the candidate SNP was rare. For the simulation scenarios using the Balding-Nichols model, we set the minor allele frequency of the candidate SNP to be 0.01 in population 1 and either 0.05 or 0.1 in population 2. The full sample consisted of an equal proportion from each subpopulation. For the scenarios where genome-wide data was simulated using the 1,000 Genomes data, we set the minor allele frequency to be *q* = (0.01, 0.02, 0.05, 0.1) for the Italian (TSI), Spanish (IBS), UK (GBR) and Finnish (FIN) samples, respectively. No other simulation settings were changed.

## 3. Results

### 3.1. False Positive Rate Under the Different Sampling Schemes

We estimated the false positive rate for the different combinations of population mixing proportion (ω_*i*_), *A* allele frequency (*p*_1_, *p*_2_) and mean phenotype value (μ_1_) listed in Table 1. As similar conclusions can be drawn from the different scenarios run, we restrict our attention to the sets of simulations having population mixing proportions ω_1_ = 0.3 and ω_1_ = 0.5 and phenotypic mean μ_1_ = 0.1. Results for the other values of ω_1_ and μ_1_ = 0.2 can be found in Supplementary Tables 1–5.

**Figure 1:**
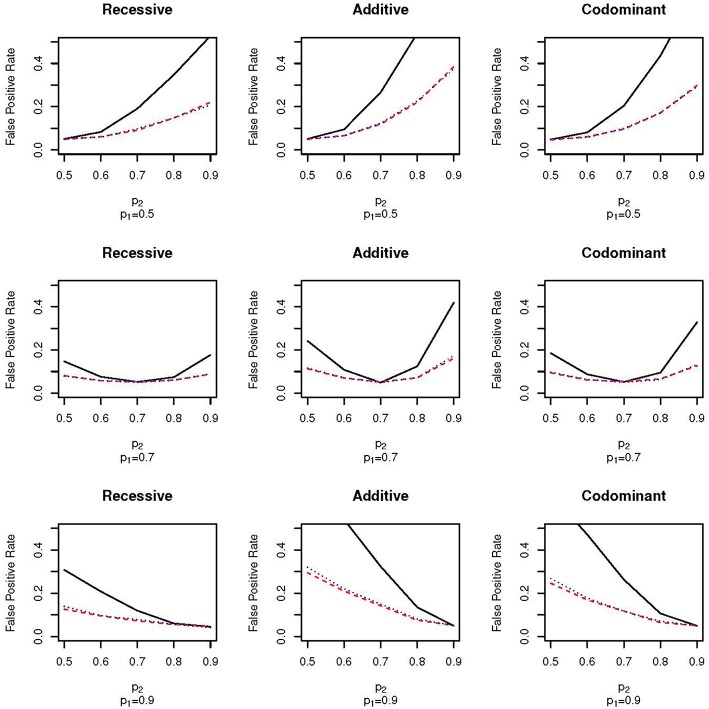
False positive proportion for scenarios with **ω_1_ = 0.3** and μ = 0.1. The solid, black line gives the false positive rate under EPS. The dashed, red line gives the false positive rate under random sampling. The dotted, blue line gives the rate under case-control sampling. The first column corresponds to the recessive test, the second column corresponds to the additive test and the third column corresponds do the codominant test. The first row corresponds to simulations with *p*_1_ = 0.5, the second row corresponds to simulations with *p*_1_ = 0.7 and the third row corresponds to simulations with *p*_1_ = 0.9.

Figure 1 shows the false positive proportions for the nine combinations of *p*_1_ and *p*_2_ run when μ_1_ = 0.1 and ω_1_ = 0.3 (that is, the mean in population 1 is 0.1, the mean in population 2 is –0.1, the proportion of the full sample from population 1 is 0.3 and the proportion of the full sample from population 2 is 0.7). For clarity, we have only plotted results with *p*_1_ = 0.5, 0.7 and 0.9; the false positive proportions for the other values of *p*_1_ can be found in Supplementary Table 1. First, as expected, there is no increase in false positive rate when *p*_1_ = *p*_2_; the false positive rate equals the nominal type 1 error rate of α = 0.05. As the difference in allele frequency between the two populations increases, the false positive rate also increases for all sampling schemes. However, the false positive rate increases much faster for the EPS design than for the random sampling design. For example, for the additive test with *p*_1_ = 0.5 and *p*_2_ = 0.7, the false positive rate for the EPS design is approximately two times higher than for the random sampling design (0.28 vs. 0.12; Figure 1, top row, second column). Even with small differences between the allele frequencies, the false positive rates of the EPS design are higher than for random sampling. For example, for the additive test with *p*_1_ = 0.7 and *p*_2_ = 0.8 the false positive rates are 0.12 and 0.07 for the EPS and random sampling designs, respectively (Figure 1, second row, second column). Under the case-control type sampling, the false positive rates are very close to those found under random sampling.

**Figure 2:**
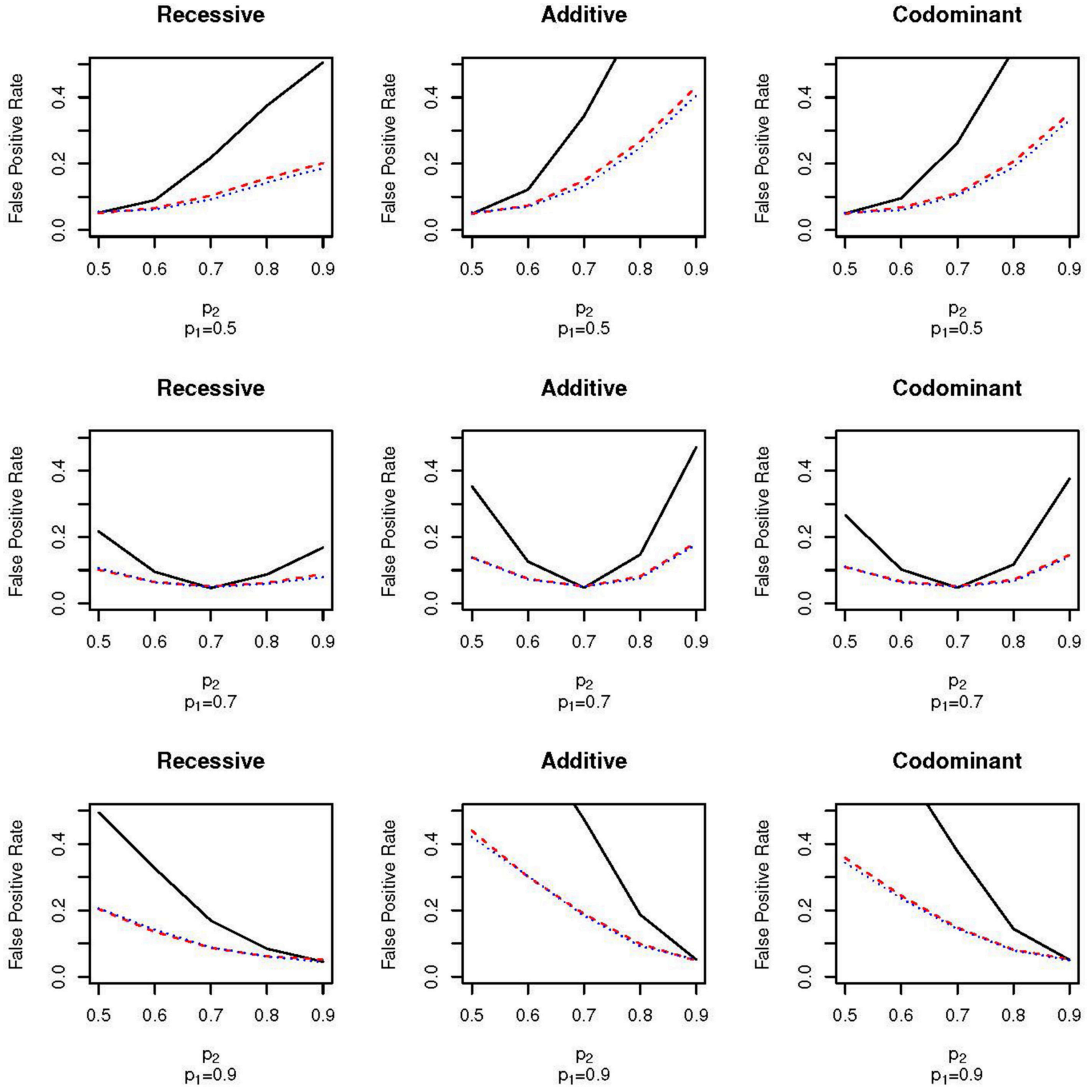
False positive proportion for scenarios with **ω_1_ = 0.5** and μ = 0.1. The solid, black line gives the false positive rate under EPS. The dashed, red line gives the false positive rate under random sampling. The dotted, blue line gives the rate under case-control sampling. The first column corresponds to the recessive test, the second column corresponds to the additive test and the third column corresponds do the codominant test. The first row corresponds to simulations with *p*_1_ = 0.5, the second row corresponds to simulations with *p*_1_ = 0.7 and the third row corresponds to simulations with *p*_1_ = 0.9.

Figure 2 shows the false positive proportions for the nine simulations run with μ_1_ = 0.1 and ω_1_ = 0.5; that is, an equal proportion of the full sample comes from each of the two subpopulations. The equal mixture presents the worst-case scenario for population stratification since neither of the two populations dominate the sample. As expected, the false positive rates for both EPS and random sampling are higher than when ω_1_ = 0.3. For example, for the codominant model with *p*_1_ = 0.9 and *p*_2_ = 0.7 the false positive rate of EPS is 0.26 when ω_1_ = 0.3 (Figure 1, bottom row, third column) and 0.38 when ω_2_ = 0.5 (Figure 2, bottom row, third column). We again see that the false positive rate increases much faster for EPS than for random sampling and case-control sampling; the rate is typically two times higher for EPS.

The pattern of false positive rates shown in both Figures 1, 2 are the same for the three different association tests. However, the false positive rate for the recessive tests are lowest while the false positive rate for the additive tests are the highest. The lower false positive rates for the recessive group may be explained by lower counts in the recessive genotype category.

The results described above show an increase in false positive rate for a range of population mixing proportions, means and major allele frequencies. One might wonder, however, whether the range of values are representative of real human populations. We therefore also ran a simulation with parameter settings motivated by real data from two European populations (Italy and France). Table 2 shows results with various values for population mixing proportion. Even for the allele frequency difference and phenotype distribution difference observed within these two European populations, we observe that the false positive rate is inflated to values that would lead to questionable association results. Even with only 20% of the sample from one of the two populations and the lower-powered recessive test, the false positive rate for EPS is 0.16 while the rate for random sampling is 0.08.

**Table 2:** Estimated false positive proportions using parameter settings from Italy and France data.

**Population proportion**		**EPS**			**Random sampling**		
**Italy**	**France**	**Recessive**	**Additive**	**Codominant**	**Recessive**	**Additive**	**Codominant**
0.20	0.80	0.16	0.29	0.23	0.08	0.13	0.10
0.30	0.70	0.24	0.44	0.34	0.11	0.18	0.14
0.40	0.60	0.30	0.53	0.43	0.13	0.21	0.16
0.50	0.50	0.34	0.58	0.47	0.15	0.25	0.19
0.60	0.40	0.33	0.55	0.45	0.14	0.23	0.17
0.70	0.30	0.29	0.47	0.37	0.14	0.19	0.15
0.80	0.20	0.20	0.31	0.24	0.10	0.13	0.11

The high false positive rate was also observed when the candidate SNP was rare in one population and either low frequency or common in the second population (Supplementary Table 6). For example, when ω_1_ = 0.3, μ = 0.1 and the candidate SNP MAFs were 0.01 and 0.05, respectively, the type 1 error rate was approximately 0.1 for the EPS sample and 0.06–0.07 for the random and “case control" sampling (Supplementary Table 6, second row). As seen with the common candidate SNP scenarios, the false positive rate increases faster with the EPS design than with the other two designs. Note that when there is no confounding due to population stratification, the codominant test appears to be conservative, with a type 1 error rate of 0.03. This is likely due to the fact that the expected counts in the least frequent genotype category would be very small with such low MAFs.

Finally, increasing the sample size does not bring the false positive rate back down to the nominal level. We simulated scenarios under mild population stratification conditions (ω_1_ = 0.1, μ = 0.1, *p*_1_ = 0.7 and *p*_2_ = 0.6) and examined the effect of increasing the cohort sample size from which the extremes are drawn from 5,000 to 10,000, 20,000, and 50,000. The corresponding subsample sizes were 2,000, 4,000, and 10,000, respectively. As the size of the subsample increases, the estimated false positive rate also increases (Table 3). For example, when the subsample consists of 1,000 from each extreme, the false positive rate is about 0.06 under EPS sampling (Table 3, first row). When the subsample consists of 5000 from each extreme, the false positive rate is between 0.11 and 0.14 (Table 3, third row). Therefore, increasing the sample size increases the severity of the problem. Note that this phenomenon also occurs when an equal-sized random sample is taken; however, the false positive rate increases more slowly. For example, when the subsample size is 10,000, the false positive rate is estimated to be 0.07–0.08 (Table 3, third row).

**Table 3:** Effect of false positive rate due to confounding when sample size is increased.

**Sample size**		**EPS**			**Random sampling**		
**Cohort**	**Subsample**	**Recessive**	**Additive**	**Codominant**	**Recessive**	**Additive**	**Codominant**
10,000	2,000	0.06	0.07	0.06	0.05	0.05	0.05
20,000	4,000	0.08	0.09	0.07	0.06	0.06	0.06
50,000	10,000	0.13	0.14	0.11	0.07	0.08	0.07

*Simulations were run with μ_1_ = 0.1, ω_1_ = 0.1, p_1_ = 0.7 and p_2_ = 0.6. The size of the subsample is 2n = 2 × 0.1 × N, where N is the cohort size*.

### 3.2. False Positive Rate After PC-Based Ancestry Correction

We estimated the false positive rate after correcting for ancestry using PCs. We considered extreme population stratification scenarios; if the correction works for the most extreme settings, then we would expect it to work well when population stratification is less extreme. We first generated ancestry data using the Balding-Nichols model and a 0.4 difference in allele frequency for the candidate SNP between populations. Results with and without the PC-based correction for a range of values of μ_1_ are shown in Table 4. As we increase the difference in the mean between the two groups from 0.2 standard deviation units to 0.4 standard deviation units, the false positive rate without correction approaches 100%. That is, we are very likely to declare the association significant even though the candidate SNP is not truly causal. However, even under these extreme population stratification scenarios, the PC-based correction controls the false positive rate so that it is close to the nominal value of α = 0.05.

**Table 4:** Estimated false positive rates before and after adjustment using the top five principal components.

		**Logistic regression (Unadjusted)**		**Logistic regression (PC-adjusted)**	
**μ_1_**	**μ_2_**	**Codominant**	**Additive**	**Codominant**	**Additive**
0.1	–0.1	0.819	0.880	0.056	0.056
0.15	–0.15	0.993	0.996	0.045	0.0495
0.175	–0.175	0.9995	0.9995	0.049	0.055
0.2	–0.2	1	1	0.059	0.057

*Logistic regression was used to test the association with the putative disease locus using either a codominant or additive genetic model*.

Results with and without the PC-based correction for the case of candidate SNPs with a rare allele are shown in Table 5. As seen with the common variant scenarios, the false positive rate increases as both the differences in phenotype means and differences in MAF (*q*) increases between the two populations. With a difference of close to 0.1 between the two MAFs, we observed a slight increase in the type 1 error rate above the 0.05 level. We therefore increased the number of simulations from 2,000 to 5,000 in order to estimate the rate more precisely. However, the type 1 error rate remains slightly elevated, particularly for the codominant model. For example, the type 1 error rate is estimated to be 0.068 under the codominant analysis model with *q*_1_ = 0.01, *q*_2_ = 0.1 and phenotype means of μ_1_ = 0.1 and μ_2_ = −0.1.

**Table 5:** Estimated false positive rates before and after adjustment using the top five principal components for the rare variant case.

				**Logistic regression (Unadjusted)**		**Logistic regression (PC-adjusted)**	
***q*_1_**	***q*_2_**	**μ_1_**	**μ_2_**	**Codominant**	**Additive**	**Codominant**	**Additive**
0.01	0.05	0.1	–0.1	0.124	0.143	0.044	0.048
0.01	0.1	0.1	–0.1	0.2788	0.339	0.068	0.055
0.01	0.05	0.2	–0.2	0.385	0.437	0.043	0.047
0.01	0.1	0.2	–0.2	0.7718	0.845	0.064	0.054

*Logistic regression was used to test the association with the putative disease locus using either a codominant or additive genetic model*.

The Balding-Nichols model generates data so that each individual SNP only has a small allele frequency difference between populations. In real data, some variants have quite different allele frequencies between populations while others have little differences due to the demographic forces that shaped the genome of human populations. To better model real genetic data, we used 1,000 Genomes data on approximately 150,000 randomly sampled common SNPs and four European populations to model our allele frequencies for generating genotype data. Results were similar to those seen under the Balding-Nichols model: the false positive rate was approximately 0.4 without a PC adjustment and close to the nominal rate of 0.05 with a PC adjustment. The results were the same even when the candidate SNP was rare in one subpopulation; the false positive rate with the PC-based correction was approximately 0.05.

## 4. Discussion

In this work, we have shown that the increased power of the EPS design comes at a cost of a greatly inflated false positive rate due to confounding by population stratification. Although we showed that the other designs also have inflated false positive rates, the EPS design was the most severely inflated. We also observed false positive rates that were twice the specified type 1 error rate of 0.05 even with parameter values taken to be similar to what would be observed in a European sample. This implies that even stratifying analyses by continental population might not be enough for controlling the type 1 error rate if an EPS design is used. Fortunately, we demonstrated that for common variants even under extreme population stratification a PC-based correction using a large sample of randomly selected SNPs throughout the genome is sufficient in bringing the false positive rate down to the specified value (typically 0.05). We therefore recommend that if the EPS design is used then an ancestry correction must be included in the analysis. In addition, although we focused on population stratification, theoretically our results would apply to any confounding variable.

A difficulty with recommending ancestry correction based on genomic data is that the EPS design is often proposed as a cost cutting measure when new technologies are introduced. Addressing population stratification could therefore increase study costs. For example, the study design might involve only sequencing selected genes or pathways. If sequence data were only available on the selected genes, then it would not be possible to adjust for ancestry in the analysis since many of the genetic variants could be hypothesized to have a true association with the trait. Additional genotyping of a large number of phenotypically neutral markers throughout the genome would need to be included in order to account for population stratification.

The inflation was only made worse by increasing the sample size. Although this might seem counter-intuitive it has a simple explanation that has also been noted elsewhere (Devlin et al., 2001). Basically, the confounding variable causes a true difference in candidate SNP allele frequency between the upper and lower extreme groups. Any aspect of the study design that increases the power will therefore also increase the probability that this true difference is detected. Augmenting the sample size increases the power and therefore increases the probability that the true, but uninteresting, difference is detected. Similarly, since the EPS design itself increases power, it also increases the probability of a false rejection.

There are some limitations with our work. First, in illustrating the inflated false positive rate, we simulated a cohort that consisted of only two subpopulations. This allowed a simple quantification of the inflation, but may not be representative of real samples from human populations, which may include many subpopulations and admixture. In addition, cohorts collected today might contain several subpopulations that are less genetically differentiated (low *F*_*st*_ values) than in our simulations. For this reason, we presented the false positive rate for EPS in relation to the rate for random and case-control sampling. Since genetic epidemiologists are concerned about confounding from population stratification even for these other designs (i.e., for case control sampling and for quantitative traits), our work suggests that the problem is even worse for the EPS design even if the actual values do not reflect population stratification in real populations.

A second limitation is that we used the Balding-Nichols model to simulate variants to be used for ancestry estimation, which also may not reflect real human data. With this model, all allele frequencies differ between populations, but the actual difference for a given SNP is actually quite small. With real human data, some variants are known to have allele frequencies that differ substantially between populations (see Kosoy et al. (2009) for example), while other variants have similar frequency values. We therefore also simulated genotypes using 1,000 Genomes genotype data as our reference population; however, the number of individuals available from any one European population is small, which could limit the genetic diversity of our reference distribution.

Third, our investigation of confounding with rare variants was limited. Using logistic regression modeling, we observed a slight inflation of the type 1 error even after ancestry correction when genotype data were simulated using the Balding-Nichols model. However, logistic regression may not be the most powerful analysis strategy for rare variants and so higher inflation might be observed with statistical approaches designed for rare variants. Since our model simulations included only a single candidate SNP, we could not evaluate any rare variant methods, such as SKAT (Wu et al., 2011), which can be modified to correct for ancestry (Luo et al., 2018), or evaluate any ancestry correction methods designed for rare variants, such as Sha et al. (2016). Given the slight false positive rate inflation that we saw even after including PCs in the logistic regression model (Balding-Nichols simulation), a more in-depth exploration of PC-based corrections under EPS when there are rare variants is needed.

Many approaches have been developed for analyzing the continuous trait data while accounting for the extreme sampling (for example, Barnett et al., 2013; Lin et al., 2013). Although we did not include any of these approaches in our comparison, we would expect similar results as the increase in false positive rate in our study is due to confounding from ancestry rather than bias due to an inappropriate analysis of selected samples. As approaches that account for selected sampling are typically based on a linear model and can include covariates, researchers can also include ancestry PCs using these methods.

Evaluation of mixed model approaches was beyond the scope of this work. First, the popular linear mixed model is not an appropriate method for analyzing the data as the phenotype is not quantitative. If we erroneously analyze the quantitative phenotypes without accounting for the selected sampling, we expect biased estimation, as was demonstrated by Lin et al. (2013) with a linear model. The linear mixed model would need to be adapted for the sampling design—for example, using an approach like Barnett et al. (2013)—and specialized software would need to be developed. Recently, generalized linear mixed models have been developed for substructure correction (Chen et al., 2016). However, Luo et al. (2018) show in a comparison of a PC vs. mixed model variance component correction for substructure that the PC approach was comparable to the variance component approach for detecting rare variants except under admixture. We therefore again expect the mixed model approach to correcting for population substructure will also adequately control the type 1 error rate.

## Ethics Statement

We have used publicly-available human genotype data from the 1,000 Genomes Project to determine simulation parameters (allele frequencies) for our study. The 1,000 Genomes data is made available according to the Fort Lauderdale Agreement (more information here: http://www.internationalgenome.org/faq/do-i-need-permission-use-1000-genomes-data-my-own-scientific-research/.

## Author Contributions

MP and KB both contributed to the conception and design of the study, performed the statistical analysis, wrote and revised the manuscript, read and approved the submitted version.

### Conflict of Interest Statement

The authors declare that the research was conducted in the absence of any commercial or financial relationships that could be construed as a potential conflict of interest.
