# Supplementary material for: A Cautionary Note on the Effects of Population Stratification Under an Extreme Phenotype Sampling Design
Source: Front Genet. 2019 May 3;10:398. doi: 10.3389/fgene.2019.00398 (PMC6509877; doi:10.3389/fgene.2019.00398)
Supplement: Supplementary file 1 [file Data_Sheet_1.pdf]

Supplementary Material

FALSE POSITIVE RATE FOR THE EPS DESIGN

For the EPS design, we assume that we have phenotype values measured on a cohort of size N and that we subsample only those in the top and bottom 10% of the phenotype distribution. To do the sampling, let the lower and upper quantiles of the mixture distribution be q_l and q_u; that is,

$$0.1 = F(q_l); \quad 0.9 = F(q_u)$$

where F is given in equation (2). For particular values of the population mixing parameter, ω_i, and the subpopulation phenotype means, these quantiles are easily computed in R using a trial-and-error approach. That is, we compute $F(y)$ for multiple values of y and find the value of y where $F(y) \approx 0.1$; this value is q_l. A similar procedure is used to find q_u. Alternatively, a root finding approach such as the bisection method could be used.

Given q_l and q_u, the proportion of the lower tail group that originates from the ith subpopulation, $i = 1, 2$, is found as follows:

$$\begin{aligned} p_{i|l} = \Pr(C = i|Y \le q_l) &= \frac{\Pr(C = i, Y \le q_l)}{\Pr(Y \le q_l)} \\ &= \frac{\Pr(Y \le q_l|C = i)\Pr(C = i)}{\Pr(Y \le q_l)} \\ &= \frac{w_i}{0.1}\Phi\Big(\frac{q_l - \mu_i}{\sigma}\Big) \end{aligned}$$

where C denotes subpopulation group or cluster. Since we are considering only two subpopulations $p_{2|l} = 1 - p_{1|l}$. Similarly,

$$p_{i|u} = \Pr(C = i|Y \ge q_u) = \frac{w_i}{0.1}(1 - \Phi\Big(\frac{q_u - \mu_i}{\sigma}\Big))$$

and $p_{2|u} = 1 - p_{1|u}$.

We have assumed independence between the genotype and phenotype, conditional on population membership. Therefore, the genotype frequencies within each extreme group reflect the frequencies of the underlying population. Assuming Hardy-Weinberg Equilibrium within subpopulation, we find

$$\begin{aligned} p_{AA|l} = \Pr(g = AA|Y \le q_l) &= \Pr(g = AA|C = 1, Y \le q_l)\Pr(C = 1|Y \le q_l) \\ &\quad + \Pr(g = AA|C = 2, Y \le q_l)\Pr(C = 2|Y \le q_l) \\ &= \Pr(g = AA|C = 1)p_{1|l} + \Pr(g = AA|C = 2)p_{2|l} \\ &= \Pr(g = AA|C = 1)p_{1|l} + \Pr(g = AA|C = 2)(1 - p_{1|l}) \\ &= p_1^2 p_{1|l} + p_2^2(1 - p_{1|l}) \end{aligned}$$

where p_1 and p_2 are the frequency of the 'A' allele in population 1 and 2, respectively. Similarly, we can show that:

$$p_{Aa|l} = \Pr(g = Aa|Y \leq q_l) = 2p_1(1-p_1)p_{1|l} + 2p_2(1-p_2)(1-p_{1|l}),$$

$$p_{aa|l} = \Pr(g = aa|Y \leq q_l) = (1-p_1)^2 p_{1|l} + (1-p_2)^2(1-p_{1,l}),$$

$$p_{AA|u} = \Pr(g = Aa|Y \geq q_u) = p_1^2 p_{1|u} + p_2^2(1-p_{1|u}),$$

$$p_{Aa|u} = \Pr(g = Aa|Y \geq q_u) = 2p_1(1-p_1)p_{1|u} + 2p_2(1-p_2)(1-p_{1|u}) \text{ and}$$

$$p_{aa|u} = \Pr(g = aa|Y \geq q_u) = (1-p_1)^2 p_{1|u} + (1-p_2)^2(1-p_{1|u}).$$

These probabilities are summarized in the following table:

	Extreme Group	
Genotype	l	u
AA	$p_{AA\|l}$	$p_{AA\|u}$
Aa	$p_{Aa\|l}$	$p_{Aa\|u}$
aa	$p_{aa\|l}$	$p_{aa\|u}$
Total	1	1

Under a dominant or recessive model, the probabilities in adjacent rows are summed. For example, a recessive 'a' (or a dominant 'A') would yield the conditional probabilities:

	Extreme	
Genotype	l	u
AA or Aa	$p_{AA\|l} + p_{Aa\|l}$	$p_{AA\|u} + p_{Aa\|u}$
aa	$p_{aa\|l}$	$p_{aa\|u}$
Total	1	1

Finally, to test for differences in allele frequency between the two groups (assuming HWE), we compute the following conditional probabilities:

	l	u
A	$p_{AA\|l} + 1/2p_{Aa\|l}$	$p_{AA\|u} + 1/2p_{Aa\|u}$
a	$p_{aa\|l} + 1/2p_{Aa\|l}$	$p_{aa\|u} + 1/2p_{Aa\|u}$
	1	1

We are interested in whether the false positive rate of a test of the association of genotype with phenotype is inflated under extreme phenotype sampling. We can test this by determining the probability that the statistical test corresponding to the hypothesized disease model is rejected given the conditional probabilities in the tables above. For illustration, consider testing that the 'a' allele has the same frequency in the lower and upper group (second row of the third table). Let the true 'a' allele probabilities be p_l and p_u in the lower and upper extreme groups, respectively. To test:

$$H_0 : p_l = p_u = \pi \text{ vs } H_1 : p_l \neq p_u$$

we can use the two-sample test of equal proportions

$$Z = \frac{\hat{p_u} - \hat{p_l}}{\sqrt{var(\hat{p}_u - \hat{p}_l)}}$$

where

$$var(\hat{p}_u - \hat{p}_l) = \frac{p_u(1-p_u)}{n} + \frac{p_l(1-p_l)}{n}.$$

and $n = Nx$. Note that when there is confounding p_l and p_u will not be equal. Therefore, to compute the probability of a false rejection, we compute the probability of rejecting the hypothesis for the values of p_l and p_u from the third table. This probability can be found using a formula for the power of a two sample test of proportions (see **?** for example):

$$1 - \Phi\Big(z_{\alpha/2} - \frac{|p_l - p_u|}{\sqrt{\frac{p_u(1-p_u)}{n} + \frac{p_l(1-p_l)}{n}}}\Big) + \Phi\Big(-z_{\alpha/2} - \frac{|p_l - p_u|}{\sqrt{\frac{p_u(1-p_u)}{n} + \frac{p_l(1-p_l)}{n}}}\Big). \quad \text{(S1)}$$

For a codominant model, a power formula for a chi-square test can be used to determine the false positive rate under confounding.

As illustration, assume that $\mu_1 = -0.1$, $\mu_2 = 0.1$, $\sigma = 1$, $p_1 = 0.7$, $p_2 = 0.5$ and $\omega_1 = \omega_2 = 0.5$. Then

$$q_l = -1.287958, \; q_u = 1.287958$$

and the conditional probabilities of genotype within lower/upper extreme groups are:

	Extreme	
Genotype	l	u
AA	0.39091	0.34909
Aa	0.45303	0.46697
aa	0.15606	0.18394

The ‘a’ allele frequency is about 0.38 and 0.42 in the lower and upper groups, respectively. Using the given formula, we would compute the probability of rejection to be about 0.36 for the additive test. These values are quite close to those computed using simulation (Figure 2, second row, left-most point of the black line and Supplementary Table 3).

SUPPLEMENTARY TABLES

Supplementary Tables 1-5 are available in the file Table 2.XLSX.

Table 1 - Estimated probability of a false positive when the proportion from population 1 and 2 is 0.3 and 0.7, respectively

Table 2 - Estimated probability of a false positive when the proportion from population 1 and 2 is 0.4 and 0.6, respectively

Table 3 - Estimated probability of a false positive when the proportion from population 1 and 2 is 0.5 and 0.5, respectively

Table 4 - Estimated probability of a false positive when the proportion from population 1 and 2 is 0.6 and 0.4, respectively

Table 5 - Estimated probability of a false positive when the proportion from population 1 and 2 is 0.7 and 0.3, respectively

Table 6 - Estimated probability of a false positive under the ”rare” variant scenarios
